# Review of “The Encyclopedia of medical & veterinary entomology” by Richard Russell, Domenico Otranto & Richard Wall

**DOI:** 10.1186/1756-3305-6-339

**Published:** 2013-12-04

**Authors:** Basil D Brooke

**Affiliations:** 1 Centre for Opportunistic, Tropical & Hospital Infections, National Institute for Communicable Diseases, Private Bag X4 Sandringham, Johannesburg, 2131, South Africa; 2 Wits Research Institute for Malaria, School of Pathology, Faculty of Health Sciences, University of the Witwatersrand, Johannesburg, South Africa

## Book details

Russell RC, Otranto D, Wall RL: *Encyclopedia of Medical & Veterinary Entomology.* CAB International; 2013. 429 pages. ISBN-13: 978 1 78064 037 2.

## Review

The Encyclopedia of Medical and Veterinary Entomology is an updated version of the 1984 publication by Prof. Douglas Kettle. Kettle’s original work, the second edition published in 1995 and the latest edition by Russell, Otranto and Wall, are three of a handful of texts covering this broad field. The latest edition is presented in an encyclopedia format, and is intended to be used as a resource for students, researchers and medical and veterinary professionals. I was therefore especially pleased to review the latest edition, as it afforded me an opportunity to revisit the essential facts concerning insects of medical importance, something I haven’t done since I was an entry-level post-graduate student.

Just by looking at the table of contents, the reader is immediately reminded or introduced to the extent of medical entomology, to the number and diversity of arthropod taxa that are of medical and veterinary significance. Most usefully, the book begins with a two-part section which introduces the medically important taxa and gives the contexts to their medical significance: as pathogen vectors, or as parasites, or as the stingers or biters that envenomate and elicit powerful allergic reactions, or as carrion feeders of forensic interest. Of particular importance is the item on emerging issues associated with these taxa and how changes in their geographical and temporal occurrences are affecting vector-borne disease incidences globally.

The book is arranged alphabetically and therefore begins with a chapter on ants. The text of this section is undoubtedly informative and provides all the basic information a student in this field needs. However, the text could have been enriched by more illustration. Unfortunately there is only one colour plate showing a myrmecine ant. Appropriate line drawings showing basic hymenopteran morphology and important morphological differences between medically important sub-families would have been informative. It is also a pity that the text is not directly referenced as opposed to providing selected bibliographies for each chapter. For example, the text describing pharaoh ants that have been implicated in the mechanical transmission of pathogens in hospitals is potentially important to researchers making an immediate link to the source material useful.

The comments concerning illustration are relevant to many chapters in the book. For example, a picture of a bed bug infestation would have been useful, as would an illustration showing the basic differences between staphylinid (rove) and meloid beetles, especially in terms of their fore-wing morphology. Illustrations of disease transmission cycles showing hosts, intermediate hosts and vectors where appropriate, such as Chagas disease and malaria, would also have been useful. Lastly, a glossary of specialised terms would have been helpful to students.

These criticisms, if they are that, do not detract from the wealth of information in the text. I especially enjoyed the chapter on fleas which mentions the disturbing fact that a third plague pandemic is currently underway. The threat of plague is all too easily forgotten and it is worth reminding health professionals that ongoing plague surveillance in vulnerable regions is important. The authors did not include the possibility of using dog sera as a plague surveillance tool, but this small omission merely highlights the fact that the field of medical and veterinary entomology is burgeoning in information and practice. This applies to the ongoing use of microfilaricides such as ivermectin for the treatment and control of human onchocerciasis, and to recent reports of resistance to ivermectin in parts of Africa.

Resistance to anti-pathogen compounds and chemically based vector control interventions is of fundamental importance in the field of medical and veterinary entomology and has become especially pertinent in malaria control. Insecticide resistance in malaria vector mosquito populations has become so widespread that the phrases ‘malaria vector control’ and ‘insecticide resistance management’ are almost interchangeable. Unfortunately, the issue of resistance is only lightly touched upon in the section covering the control of malaria vectors, and the numerous alternative biological and genetic methods that are being intensively evaluated are not mentioned. Nevertheless, the chapter on mosquitoes and the pathogens they transmit is otherwise comprehensive and gives a good perspective of what is arguably the most important insect family (Culicidae) in terms of disease transmission.

In conclusion, any student, researcher or health professional requiring a compendium of arthropod taxa of medical importance, the numerous pathogens they transmit, their biological and morphological characteristics, and the methods used to control them, will find this encyclopedia informative, sufficiently detailed in most instances, and easy to use. I am especially pleased to have acquired a copy.

## Competing interests

The author declares that he has no competing interests.

